# Dose and time responses of vitamin D biomarkers to monthly vitamin D_3_ supplementation in overweight/obese African Americans with suboptimal vitamin d status: a placebo controlled randomized clinical trial

**DOI:** 10.1186/s40608-015-0056-2

**Published:** 2015-07-04

**Authors:** Jigar Bhagatwala, Haidong Zhu, Samip J. Parikh, De-Huang Guo, Ishita Kotak, Ying Huang, Robyn Havens, Michael Pham, Eric Afari, Susan Kim, Christopher Cutler, Norman K. Pollock, Yutong Dong, Anas Raed, Yanbin Dong

**Affiliations:** Georgia Prevention Institute, Medical College of Georgia, Georgia Regents University, Building HS-1640, Augusta, 30912-3715 GA USA; Department of Internal Medicine, Medical College of Georgia, Georgia Regents University, Augusta, GA USA; College of Nursing, Georgia Regents University, Augusta, GA USA; College of Dental Medicine, Georgia Regents University, Augusta, GA USA

**Keywords:** Monthly vitamin D, Overweight/obese, African Americans, Dose–response vitamin D, 4000 IU vitamin D

## Abstract

**Background:**

A critical need exists to better understand the physiological sequel of vitamin D supplementation in obese individuals and African Americans. The aim was to comprehensively evaluate dose- and time-responses of a panel of vitamin D biomarkers to vitamin D supplements in this population.

**Methods:**

We conducted a 16-week randomized, double-blinded, and placebo-controlled clinical trial. Seventy overweight/obese African Americans (age 13–45 years, 84 % females) with 25-hydroxyvitamin D [25(OH)D] concentrations ≤20 ng/mL were randomly assigned to receive a supervised monthly oral vitamin D_3_ of 18,000 IU (~600 IU/day, *n* = 17), 60,000 IU (~2000 IU/day, *n* = 18), 120,000 IU (~4000 IU/day, *n* = 18), or placebo (*n* = 17).

**Results:**

There were significant dose- and time-responses of circulating 25(OH)D, 1,25-dihydroxyvitamin D [1,25(OH)_2_D], and intact parathyroid hormone (iPTH), but not fibroblast growth factor-23 (FGF-23), phosphorus and urine calcium to the vitamin D supplements. The mean 25(OH)D concentrations in the 2000 IU and 4000 IU groups reached ≥30 ng/mL as early as 8-weeks and remained at similar level at 16-weeks. The increase of 25(OH)D was significantly higher in the 4000 IU group than all the other groups at 8-weeks. The increase of 1,25(OH)_2_D was significantly higher in the 2000 IU and 4000 IU groups than the placebo at 8-weeks. Only the 4000 IU compared to the placebo significantly reduced iPTH at 8- and 16-weeks.

**Conclusions:**

Our RCT, for the first time, comprehensively evaluated time- and dose- responses of vitamin D supplementation in overweight/obese African Americans with suboptimal vitamin D status. Circulating 25(OH)D, 1,25(OH)2D, and iPTH, but not FGF-23, phosphorus and urine calcium, respond to vitamin D supplementation in a time- and dose–response manner. By monthly dosing, 2000 IU appears to be sufficient in achieving a 25(OH)D level of 30 ng/mL in this population. However, importantly, 4000 IU, rather than 2000 IU, seems to suppress iPTH. If replicated, these data might be informative in optimizing vitamin D status and providing individualized dosing recommendation in overweight/obese African Americans.

**Trial registration:**

ClinicalTrials.gov number: NCT01583621, Registered on April 3, 2012.

## Background

African Americans as compared to their Caucasian peers have disproportionately high prevalence of suboptimal vitamin D status [1–3]. Obesity, a risk factor for poor vitamin D status, also negatively impacts 25(OH)D responses to vitamin D supplementation [3–5]. The most recent Institute of Medicine (IOM) Report identified an urgent need to conduct randomized clinical trials (RCTs) of vitamin D supplementation to clarify dose–response to intake, with a special emphasis on the individuals with excess adiposity and dark skin pigmentations [6]. Given the lack of conclusive evidence, the IOM bases its Recommended Dietary Allowance (RDA) for vitamin D on bone health, which is 600–800 IU/day corresponding to a 25(OH)D serum level of ~20 ng/mL [6]. The target 25(OH)D level is ~30 ng/mL recommended by many other experts for large swaths of the population, although it is controversial [7–10].

In an earlier placebo-controlled RCT, 208 healthy postmenopausal African American females residing in New York (~40.8°N latitude) were given a daily dose of 800 IU vitamin D plus calcium for two years [11]. The mean 25(OH)D levels remained <30 ng/mL at the end of the second year, but reached up to 35 ng/mL within three months after the dose was increased to 2000 IU. However, approximately 40 % of participants still had 25(OH)D levels between 20–30 ng/mL. The authors suggested a dose between 2800-4000 IU/day to optimize vitamin D levels in African Americans, according to the vitamin D algorithm. Gallagher and colleagues [12] supplemented 110 postmenopausal African American females with suboptimal vitamin D status living in Nebraska (~41°N latitude) and Indiana (~40°N latitude) with daily doses between 400–4800 IU vitamin D plus calcium for 12 months. The authors determined that a daily dose of 1600 IU was required to achieve the level of 25(OH)D ≥30 ng/mL. In another study of younger (25–45 years) African American (*n* = 39) and Caucasian (*n* = 90) females living in Nebraska, 400–2400 IU/day vitamin D plus calcium supplements resulted in a dose and time-responsive increase in serum 25(OH)D concentrations. The authors estimated a daily dose between 800–1600 IU in young African American females, to achieve a level of serum 25(OH)D at 20 ng/mL [12]. More recently, Ng et al., [13] supplemented 30–80 years-old 328 African Americans living in Boston (42°N latitude) with daily 1000, 2000, or 4000 IU of vitamin D plus calcium for 3 months. It was estimated that a dose of 1640 IU and 4000 IU vitamin D is desired to achieve the 25(OH)D concentrations of ≥20 ng/mL and ≥33 ng/mL, respectively. However, dose-responsive RCTs with exclusive vitamin D supplementation are lacking in young African Americans, particularly in overweight/obese individuals.

Circulating 25(OH)D is converted by the enzyme 1-α hydroxylase to 1,25-dihydroxyvitamin D [1,25(OH)_2_D], the active form of vitamin D, which is responsible for the biological actions on calcium and phosphate homeostasis. 1,25(OH)_2_D has suppressive effects on parathyroid hormone (PTH) release and the level at which 25(OH)D begins to suppress PTH has been utilized to define vitamin D sufficiency [14, 15]. Fibroblast growth factor-23 (FGF-23), a protein secreted from bone, plays a major role in the regulation of conversion of 25(OH)D to 1,25(OH)_2_D by suppressing 1-α hydroxylase. FGF-23 also enhances the expression of 24-hydroxylase, an enzyme that converts 1,25(OH)_2_D into a metabolite with much less biologic activity [16, 17]. The major regulators of FGF-23 release are 1,25(OH)_2_D, phosphate and PTH [18, 19]. However, there is paucity of dose-responsive RCTs evaluating changes of these vitamin D biomarkers to vitamin D supplementation, particularly in African Americans.

Therefore, the main objective of the current RCT was to study dose- and time-responses to vitamin D_3_ supplements of 600 IU/day (current recommended dietary allowance, RDA), 2000 IU/day (previously the tolerable upper intake level [UL]) [20] and 4000 IU/day (the new UL) on circulating 25(OH)D, 1,25(OH)_2_D, PTH, FGF-23, phosphorus, and urinary calcium in apparently healthy young overweight/obese African Americans with suboptimal vitamin D status residing in Southeastern United States. The present RCT undertook a monthly dosage scheme. Monthly dosing that is expected to improve compliance compared to daily dosage is commonly practiced for vitamin D supplementation [21–23]. However, dose- and time- responses by monthly dosing are not well understood.

## Subject and methods

### Participants

In this randomized, double-blinded, placebo-controlled clinical trial (clinicaltrials.gov registration#: NCT01583621), participants were recruited from community by advertisements and by word of mouth. Inclusion criteria were self-reported African American race, age between 13–45 years, overweight/obese, no pregnancy, no lactation, no known acute or chronic illnesses, no use of any prescription medications, birth control pills, herbal, multi-vitamin or mineral supplementations, and suboptimal vitamin D status (serum 25(OH)D concentrations of ≤20 ng/mL) [6] at screening visit. Overweight/obesity was defined by body mass index (BMI) ≥25 kg/m^2^ for adults and ≥85^th^ percentile for age and sex for adolescents according to the Center of Disease Control and Prevention (CDC) criteria. The study was approved by the institutional review board (IRB) at the Georgia Regents University (GRU). Adolescents and their guardians provided informed written assent and consent, respectively; and the adults provided informed consent. A total of 129 overweight/obese African Americans living in Augusta, GA and surrounding areas were screened and 70 eligible subjects were enrolled and followed-up in the between December, 2011 and November, 2012. The trial registration on clinicaltrials.gov was delayed due to an oversight, and the authors confirm that all ongoing and related trials for this intervention are registered.

### Randomization and vitamin D_3_ dosing

**Fig. 1:**
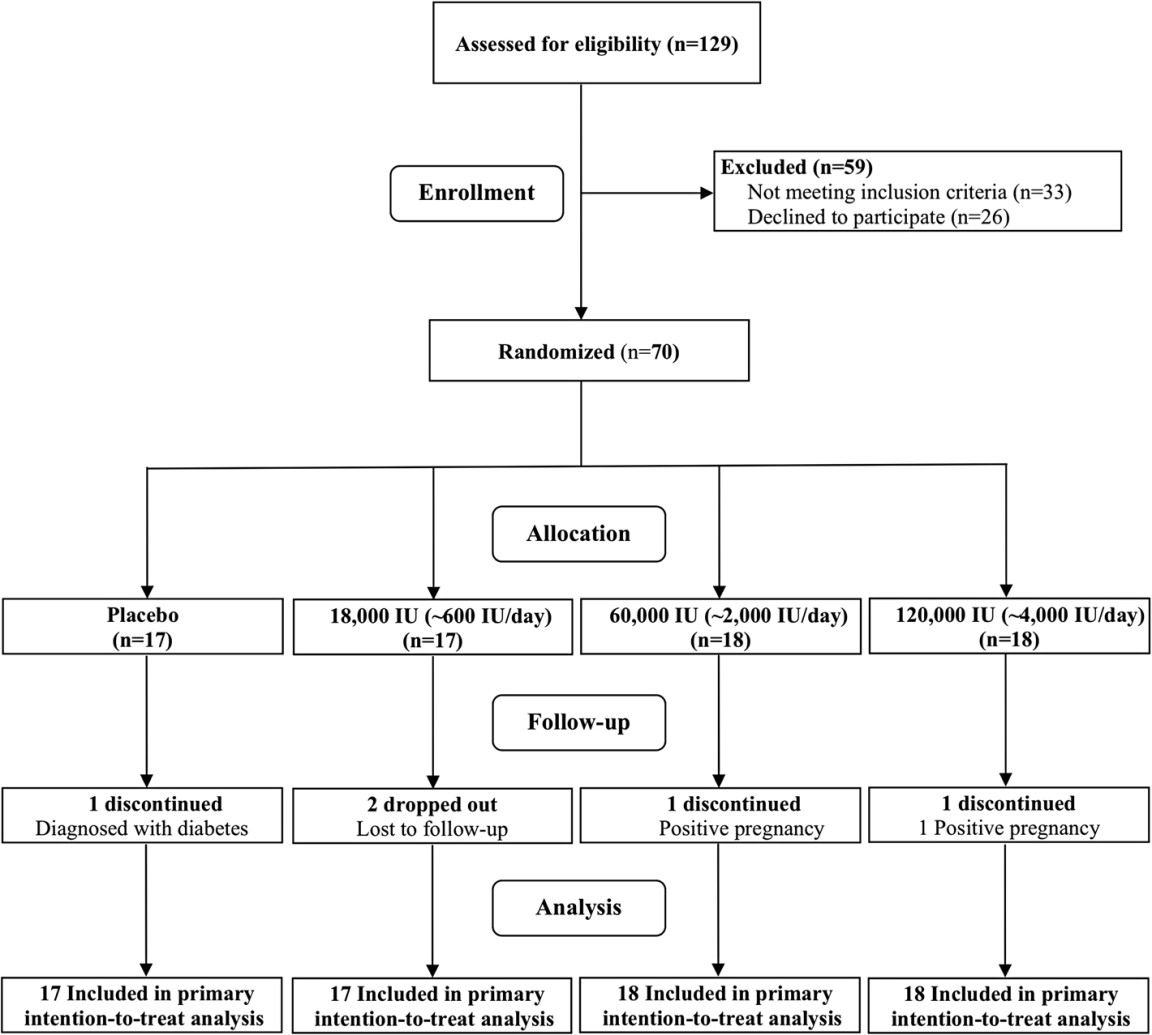
Randomization scheme and group details (CONSORT 2010 flow chart). 25(OH)D: 25-hydroxyvitamin D

As presented in Fig. 1, participants were randomly assigned to either of the following vitamin D supplement groups: 18,000 IU (equivalent to 600 IU/day), 60,000 IU (equivalent to 2000 IU/day), 120,000 IU (equivalent to 4000 IU/day) or a placebo for 16 weeks by supervised monthly dosing. The vitamin D and placebo capsules were provided by the Bio-Tech Pharmacal, Fayetteville, AR, and the GRU clinical research pharmacy generated the randomization codes and dispensed the study capsules. The GRU clinical pharmacy maintained the randomization codes until the end of the study and did not have any direct role in the data collection.

### Laboratory tests

Fasting blood samples and spot urine samples were obtained at baseline, 8- and 16-weeks, which were frozen and stored at −80 °C until assayed. Serum 25(OH)D concentrations were measured using enzyme immunoassay (Immunodiagnostic Systems, Fountain Hills, AZ). The intra- and inter-assay coefficients of variation (CV) were 5.6 and 6.6 %, respectively. Our laboratory is certified by the vitamin D external quality assessment scheme (DEQAS), an international program monitoring accuracy of 25(OH)D measurements. Plasma 1,25(OH)_2_D levels were measured using radioimmunoassay [24]. The intra- and inter-assay CVs were 9.8 % and 12.6 %, respectively. Plasma bioactive intact parathyroid hormone (iPTH) concentrations were measured using an enzyme-linked immunosorbent assay (ELISA, Immutopics, San Clemente, CA). Plasma intact FGF-23 concentrations were measured using ELISA (Immunotopics, Inc., San Clemente, CA). The intra- and inter-assay CVs were and 6.7 % and 9.8 %, respectively. Plasma phosphorus was determined using colorimetric assays kits (Point Scientific, Inc., Canton, MI). The intra- and inter-assay CVs were 6.4 % and 8.1 %, respectively. Spot urine calcium levels were measured by BioVision’s Colorimetric Calcium Assay Kits. The urine calcium normalized by urine creatinine (Ca/Cr) was used to monitor toxicity.

### Sample size estimation

Power calculations were computed based on primary outcomes 25(OH)D, 1,25(OH)_2_D, iPTH, and FGF-23. The computations are based upon the linear contrast approach for the main effect of treatment, rather than the traditional omnibus F-test followed by post hoc pairwise comparisons, because a pre-specified contrast conditioned on the ordering of responses is more powerful to detect the expected pattern, without sacrificing control of type I error. Required are assumptions concerning the difference in mean 16-week changes in the response (i.e., differences in the mean gain scores) across treatment arms, and the variance of the gain score, from which effect size is computed. Using previous data, including ours, [3, 25, 26] we pooled estimates in standard deviation (SD) of change for 25(OH)D (SD = 5.1 ng/mL), 1,25(OH)_2_D (SD = 4.0 pg/mL), iPTH (SD = 3.5 pg/mL) and FGF-23 (SD = 1.2 pg/mL), and we estimated effect sizes (i.e., Cohen’s *d*) for these outcomes [25(OH)D, *d* = 3.0; 1,25(OH)_2_D, *d* = 1.2; iPTH, *d* = 0.65; and FGF-23, *d* = 0.53] based on the assumption that the 600 IU and 2000 IU groups would have one-quarter and one-half the mean change, respectively, of that occurring in the 4000 IU group, relative to the mean change in the control group. We determined that 10–16 subjects/group would provide 81-96 % power (α = 0.05) to detect a difference in mean change of the outcome variable between groups. With a given sample size of 16 subject/group (α-level set at 0.05), the proposed study will have power of 0.81. Assuming a 10 % sample size loss due to attrition or insufficient quality of measurements, a starting sample size of 18 subjects per group will preserve power of the study design.

### Statistical analyses

All statistical analyses were performed using SPSS software (IBM Corp. Released 2011. IBM SPSS Statistics for Windows, version 21.0. Armonk, NY) and statistical significance was set at P < 0.05. Analysis of variance was used to compare group differences at baseline for normally distributed variables or by Kruskal-Wallis test, otherwise. Differences in proportions were tested by chi-square test of goodness of fit.

Repeated-measures mixed-models were used with maximum likelihood estimation in an intention-to-treat analysis of each outcome measure using all available data. Base models for each outcome measure included the fixed effects of intervention group (placebo, 600 IU/day, 2000 IU/day, or 4000 IU/day) and measurement time (baseline, 8-weeks, or 16-weeks) and their interaction. The modeled covariance structure between measurement periods was unstructured, which used all available measurements on the same subject, including those from subjects who dropped out of the study. Because significant group differences in baseline characteristics were not detected, covariates were not considered in the primary analyses. However, secondary analyses were performed to consider potential effects of age, gender, season, and BMI. The linear contrasts across the four groups of the change over time tested the dose–response effects of vitamin D supplementation, and the pairwise comparisons of changes in the outcome variables between groups were performed.

## Results

### Baseline clinical characteristics

Table 1 displays the baseline characteristics of the participants. Of 129 screened, ~75 % (96 individuals) met the inclusion criteria. A total of 70 subjects were available at baseline. The baseline serum 25(OH)D in the entire sample was 14.77 ± 0.6 ng/mL. Age, gender distribution, BMI, serum 25(OH)D, plasma iPTH, 1,25(OH)_2_D, FGF-23 and phosphorus did not differ among the groups at baseline. No side effects were observed during the study period. A total of 5 participants discontinued the study either due to loss of follow up (2 subjects), positive pregnancy test (2 subjects) or diagnosis of diabetes (1 subject).

**Table 1:** Baseline clinical characteristics

Variable	Study groups				*P*-value
	Placebo (*n* = 17)	600 IU/day (*n* = 17)	2000 IU/day (*n* = 18)	4000 IU/day (*n* = 18)	
Age (years)	27.78 ± 2.6	26.19 ± 2.5	24.38 ± 2.0	25.51 ± 2.2	0.77
Male/Female^a^	4/12	2/13	3/14	2/15	0.76
Height (cm)	164.28 ± 2.1	164.60 ± 2.0	163.84 ± 2.4	164.20 ± 1.7	0.99
Weight (kg)	98.42 ± 6.7	93.39 ± 3.7	99.16 ± 5.0	92.77 ± 4.8	0.74
BMI (kg/m^2^)	36.19 ± 2.0	34.56 ± 1.4	37.08 ± 1.9	34.42 ± 1.7	0.66
Serum 25(OH)D (ng/mL)	15.88 ± 1.4	14.00 ± 0.8	15.93 ± 1.0	13.25 ± 1.0	0.21
Plasma iPTH (pg/mL)^#^	47.32 ± 3.6	59.60 ± 7.8	50.60 ± 4.2	62.00 ± 8.8	0.32
Plasma 1,25(OH)_2_D (pg/mL)	34.04 ± 1.9	37.40 ± 1.8	35.18 ± 2.3	32.70 ± 1.1	0.46
Plasma FGF-23 (pg/mL)^#^	6.53 ± 0.9	6.92 ± 0.8	8.57 ± 1.6	6.57 ± 0.9	0.81
Plasma Phosphorus (mg/dL)^#^	3.74 ± 0.2	4.01 ± 0.3	3.61 ± 0.1	3.80 ± 0.1	0.71
Urine Ca/Cr ratio	0.08 ± 0.05	0.05 ± 0.03	0.10 ± 0.05	0.08 ± 0.05	0.09

Values are presented as mean ± s.e.m

*BMI* Body mass index, *25(OH)D* Serum 25-hydroxyvitamin D, *Plasma 1,25(OH)*_*2*_ *D* 1,25-dihydroxyvitamin D, *iPTH* Intact parathyroid hormone, *FGF-23* Fibroblast Growth Factor-23, *Ca/Cr* Calcium/creatinine ratio

^a^Group differences analyzed using chi-square test

^#^Group differences analyzed using Kruskal-Wallis test

### 25 (OH)D: dose and time responses at 8- and 16-weeks

**Fig. 2:**
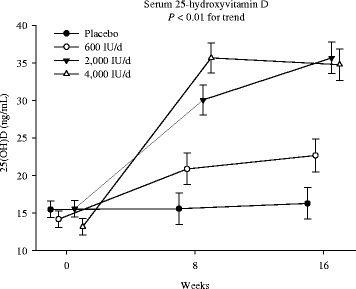
Dose- and time-responses of 25(OH)D. Intention-to-treat mixed-model repeated-measures analysis of variance of the effect of group on serum 25-hydroxyvitamin D [25(OH)D], the *P*-value in each panel indicates the test of the dose–response trend. Error bars indicate 95 % confidence intervals

There were overall group by time interactions (P < 0.01), suggesting dose- and time-dependent increases in serum 25(OH)D concentrations to the monthly vitamin D supplements (Fig. 2). In the 600 IU group, mean 25(OH)D concentrations remained ~20 ng/mL both at 8- (21.0 ± 1.0 ng/mL) and 16-weeks (22.61 ± 1.24 ng/mL). On the other hand, both 2000 IU and 4000 IU vitamin D groups raised mean 25(OH)D concentrations to the level of 30 ng/mL at 8-weeks (30.50 ± 2.1 and 35.66 ± 3.4 ng/mL, respectively), and maintained at a similar level (36.01 ± 3.1 and 34.80 ± 2.4 ng/mL, respectively) at 16-weeks.

In post-hoc group-wise comparison, the changes in 25(OH)D concentrations from baseline to 8-weeks in the 4000 IU group (22.41 ± 1.8 ng/mL) were significantly greater compared to the 2000 IU (14.56 ± 1.8 ng/mL, p < 0.01), 600 IU (7.06 ± 2.0 ng/mL, p < 0.01) and placebo (0.12 ± 1.9 ng/mL, p < 0.01) groups. At 16-weeks, however, the changes in 25(OH)D concentrations in the 4000 IU group plateaued and were similar to the changes in the 2000 IU group (21.51 ± 2.0 vs. 20.08 ± 2.0 ng/mL, *p* = 0.60). Within the 4000 IU group, 25(OH)D concentrations at 16-weeks did not differ from those at 8-weeks (34.77 ± 2.5 vs. 35.66 ± 3.4 ng/mL, *p* = 0.60). However, within the 2000 IU group, 25(OH)D concentrations at 16-weeks were significantly higher than those at 8-weeks (36.01 ± 3.1 vs. 30.50 ± 2.1 ng/mL, *p* = 0.02).

### 1,25 (OH)_2_D: dose and time responses at 8- and 16-weeks

**Fig. 3:**
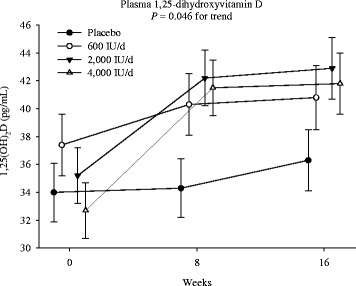
Dose- and time-responses of 1,25(OH)_2_D. Intention-to-treat mixed-model repeated-measures analysis of variance of the effect of group on plasma 1,25-dihydroxyvitamin D [1,25(OH)_2_D], the *P*-value in each panel indicates the test of the dose–response trend. Error bars indicate 95 % confidence intervals

There were overall group by time interactions (*P* = 0.046), suggesting dose- and time-dependent increases in plasma 1,25(OH)_2_D concentrations to the monthly vitamin D supplements (Fig. 3). In post-hoc group-wise comparisons, the changes from baseline to 8-weeks, but not the changes from baseline to 16-weeks, were significantly higher both in the 4000 IU and 2000 IU groups versus the placebo group. However, only the 4000 IU group showed a greater increase than the 600 IU group (8.89 ± 2.0 pg/mL vs. 2.95 ± 2.1 pg/mL, *p* = 0.04) at 8-weeks. The increases of 1,25(OH)_2_D concentrations at 16-weeks in the 4000 IU group versus the placebo or 600 IU group was marginally significant (*p* = 0.07 and 0.08, respectively). Nevertheless, the increases of 1,25(OH)_2_D in the 600 IU group were not statistically significant either at 8- or 16-weeks.

### PTH: dose and time responses at 8- and 16-weeks

**Fig. 4:**
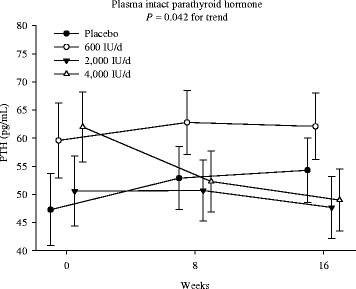
Dose- and time-responses of iPTH. Intention-to-treat mixed-model repeated-measures analysis of variance of the effect of group on plasma intact parathyroid hormone (iPTH), the *P*-value in each panel indicates the test of the dose–response trend. Error bars indicate 95 % confidence intervals

There were overall group by time interactions (*p* = 0.042), suggesting dose- and time-dependent reductions in plasma iPTH concentrations to the monthly vitamin D supplements (Fig. 4). A post-hoc group-wise comparison showed that iPTH concentrations reduced significantly from baseline only in the 4000 IU group, rather than the 600 or 2000 IU group, both at 8-weeks and 16-weeks (p < 0.05), as compared to the placebo group. The changes did not differ among the other groups either at 8- or 16-weeks.

### Plasma FGF-23, phosphorus and urine Ca/Cr ratio: dose and time responses at 8- and 16-weeks

There were not significant overall group by time interactions in FGF-23, phosphorus, and urine Ca/Cr ratio either at 8- or 16-weeks (all p > 0.05).

## Discussion

To our knowledge, this is the first dose–response vitamin D supplementation RCT comprehensively evaluating vitamin D responses in overweight/obese African Americans with suboptimal vitamin D status. Our major findings include: 1) vitamin D_3_ supplements by monthly dosing increased circulating 25(OH)D and 1,25(OH)D; and suppressed iPTH in a time- and dose-dependent manner; 2) both 2000 IU/day and 4000 IU/day, rather than 600 IU/day, achieved the 25(OH)D level of 30 ng/mL. Although 4000 IU/day was not superior to 2000 IU/day in increasing 25(OH)D concentrations, 4000 IU/day increased 25(OH)D concentrations more rapidly than 2000 IU/day by monthly dosing; 3) Perhaps more importantly, only 4000 IU/day suppressed iPTH; and 4) plasma FGF-23, phosphorus and urine calcium did not change in time- or dose- dependent manner.

### 25(OH)D

Only few RCTs have studied dose-responsiveness to vitamin D supplementation in African Americans. We report for the first time that monthly vitamin D_3_ raises serum 25(OH)D concentrations in a dose- and time-dependent manner in overweight/obese African Americans with suboptimal vitamin D status as defined by serum 25(OH)D level ≤ 20 ng/mL [6]. By monthly dosing, 2000 IU and 4000 IU vitamin D, rather than 600 IU, are both efficient in optimizing vitamin D status over 16 weeks. Of note, the current RDA of 600 IU/day is not well studied in African Americans. Previous dose–response trial in nursing home residents in Boston [27] reported that 600 IU/day vitamin D may not be adequate in achieving a 25(OHD) level of 30 ng/mL. In the elderly living in the Netherlands with suboptimal vitamin D status, a dose of 600 IU/day did not achieve the level of 30 ng/mL either by daily, weekly or monthly dosing after ~4 months [28].

One of the most interesting findings of our study was that the 25(OH)D concentrations at posttest between the 4000 IU and 2000 IU groups did not differ. The mean 25(OH)D concentrations in the 4000 IU group seemed to plateau and become similar to the 25(OH)D concentrations in the 2000 IU group. In the dose–response trial by Gallagher in post-menopausal Caucasian females with 25(OH)D concentrations below 20 ng/mL, [29] the 25(OH)D concentrations increased in a quadratic, rather than a linear pattern with an increasing vitamin D dose from 400–4800 IU/day. The 25(OH)D concentrations in their study began to plateau at the dose of ~4000 IU. In a subset of African Americans [12], the dose–response curves for 25(OH)D concentrations were linear. The authors speculated that the observed racial differences could be due to a very small number of African Americans in the 3200 and 4000 IU groups. There are few dose–response RCTs comparing 2000 IU and 4000 IU doses. Pregnant Arab females with vitamin D deficiency were previously supplemented with 400, 2000, or 4000 IU/day [30]. The 25(OH)D responses in the 2000 and the 4000 IU groups paralleled from 12–16 weeks of gestation (baseline) to 28 weeks of gestation (~16 weeks), as observed in our study. Heaney and colleagues [31] suggest that at a lower concentration of 25(OH)D, the conversion of vitamin D_3_ from oral or cutaneous intake into 25(OH)D follows first-order kinetics, which later switches to a slower, zero-order with saturation of hepatic 25-hydroxylase and the 25(OH)D levels start to plateau. The serum 25(OH)D concentration at which this change occurs was calculated to be ~35.2 ng/mL. In our study, the 4000 IU group by monthly dosing achieved mean serum 25(OH)D concentrations of 35.66 ± 3.4 ng/mL as early as 8-weeks, making it plausible that the rate of conversion into 25(OH)D might have diminished from 8-week onwards. On the other hand, the 2000 IU achieved mean serum 25(OH)D concentrations of 30.50 ± 2.1 ng/mL at 8-weeks and continued to increase to 16-weeks. Our data indicate that a dose of 2000 IU by monthly dosing may be appropriate to improve the vitamin D status up to the level of 30 ng/mL, but 4000 IU can achieve it more rapidly.

### 1,25(OH)_2_D

We demonstrated dose- and time-dependent increases in 1,25(OH)_2_D concentrations, and 1,25(OH)_2_D concentrations can be raised by 2000 or 4000 IU in a monthly dosing as early as 8 weeks. Provided the continuous increase of 1,25(OH)_2_D concentrations at 16-weeks by 4000 IU in the present study, though marginally significant, future RCTs with a larger sample size are warranted. To the best of our knowledge, there was only one previous study evaluating the dose-responsiveness of 1,25(OH)_2_D to vitamin D supplementation, in which no significant dose-dependent increases in 1,25(OH)_2_D were observed in a population of African American and Caucasian children with daily doses ranging from 400 to 4000 IU of vitamin D supplements for 12 weeks [32]. The disparities of the findings could be due to the differences in the baseline 25(OH)D levels or the frequency of dosing.

### iPTH

We observed dose- and time-responsive reductions in plasma iPTH concentrations, whereas mixed results in this regard have been reported in the literature [12, 29, 32]. The present study demonstrated that 4000 IU, rather than 600 or 2000 IU by monthly dosing, significantly reduced iPTH at 8- and 16-weeks. Our group previously reported that iPTH concentrations in response to 2000 IU/day was unchanged in African American adolescents [3] and adults [26], which is in agreement with the findings of others [33]. These findings may reinforce it that a dose higher than 2000 IU/day would be required to suppress iPTH. However, a higher daily vitamin D dose, i.e., ~5000 IU/day, modestly yet insignificantly reduced iPTH, as compared to 1000 and 2000 IU/day, respectively [33, 34]. Thus, our results might indicate the superiority of monthly dosing in suppressing iPTH.

### FGF-23 and phosphorus

To the best of our knowledge, our study is among the few to report FGF-23 and phosphorus in apparently healthy African Americans, and the first to study the dose-responsiveness to vitamin D supplementation. Of note, baseline plasma intact FGF-23 levels in our African American subjects were lower than previously reported [35, 36]. We did not observe any dose- or time-dependent changes in either FGF-23 or phosphorus. Eighteen otherwise healthy premenopausal Turkish females with serum 25(OH)D concentrations below 30 ng/mL were previously given a loading dose of 150,000 IU vitamin D_3_ followed by daily 880 IU plus 1000 mg calcium for 6 weeks [25]. At posttest, significant reductions in FGF-23 and phosphorus were observed. Another study by Turner and colleagues in 45 osteoporotic older males and females with serum 25(OH)D concentrations below 20 ng/mL administered a single loading dose of 300,000 IU vitamin D_2_ intramuscular followed by daily 800 IU of vitamin D_3_ plus 1200 mg calcium orally for 3 months [37]. Significant increases were observed in FGF-23 and phosphorus at 1, 2, and 3 months. By supplementing weekly 50,000 IU vitamin D_2_ plus calcium in individuals between 18–45 years of age with serum 25(OH)D levels below 20 ng/mL, Burnett-Bowie and colleagues reported a significant increase in FGF-23 with no changes in phosphate concentrations after 8- and 12-weeks [38]. Thus, FGF-23 and phosphate changes in response to vitamin D supplementation are inconsistent. All these observed discrepancies could be due to, 1) the various dosing regimens; or 2) the differences in assay methods, e.g., intact FGF-23 versus c-terminal FGF-23; or 3) the use of calcium with vitamin D supplementation; or 4) low baseline FGF-23 levels in our study. We also speculate that in our study the changes of the two stimulants for FGF-23, i.e. the reductions in iPTH and increases in 1,25(OH)_2_D, might have kept the FGF-23 concentrations unchanged. Last, the control of circulating concentrations of FGF-23 is a complex matter, and monthly versus daily dosing may result in fluctuations in the concentrations of circulating FGF-23 [39].

### Strengths and limitations

There are several strengths in the present study. We exclusively recruited overweight/obese African American participants with suboptimal vitamin D status. We comprehensively analyzed various vitamin D related biomarkers, and administered vitamin D supplements without calcium supplements. The doses selected in this study were based on the current RDA and UL by the IOM, [6] and monthly supervised dosing scheme was undertaken to ensure 100 % compliance. Although monthly vitamin D dosing has been previously administered, our study is the first to use a dose–response design. The limitations of our study are worth mentioning. First, the bioavailability of 25(OH)D was not included in the current study, although we only supplemented African Americans [40]. Second, the participants were recruited in different seasons. However, only individuals with suboptimal vitamin D status were recruited irrespective of the season of enrollment, and the results did not alter after adjusting for seasons. Third, the female representation was higher in our sample compared to males. However, the gender distribution was not different among groups, and the results did not differ after adjusting for gender as a potential confounder. Fourth, the objective of the current study is to study a panel of circulating vitamin D biomarkers in response to vitamin D supplementation. Skeletal and extra-skeletal functional outcomes, that are not part of this study, would deserve investigations in the future, to help to select doses and time scheme. Finally, the sample size in each group was relatively small, which emphasizes the need for larger studies.

## Conclusions

In summary, circulating 25(OH)D, 1,25(OH)_2_D, and iPTH concentrations, but not FGF-23, phosphorus and urine calcium, respond to monthly vitamin D supplementation in a time- and dose-dependent manner. Both 2000 IU and 4000 IU vitamin D in monthly dosing appear to be comparable and effective in achieving the 25(OH)D level of 30 ng/mL, but 4000 IU optimizes vitamin D status more rapidly. In monthly dosing, 4000 IU suppresses iPTH rapidly, and the suppression seems to sustain over time. The biological benefits of 4000 IU vitamin D needs to be further investigated in well-designed, larger RCTs with skeletal and extra-skeletal outcomes.

## Abbreviations

25(OH)D: 25-hydroxyvitamin D
IOM: Institute of Medicine
RCT: Randomized clinical trials
1,25(OH)_2_D: 1,25-dihydroxyvitamin D
PTH: Parathyroid hormone
FGF-23: Fibroblast growth factor-23
RDA: Recommended dietary allowance
BMI: Body mass index
IRB: Institutional review board
DEQAS: Vitamin D external quality assessment scheme
iPTH: Intact parathyroid hormone
ELISA: Enzyme-linked immunosorbent assay
SD: Standard deviation
SEM: Standard error of mean
Ca/Cr: Calcium/creatinine ratio
